# Rheological and functional characterization of gelatin and fat extracted from chicken skin for application in food technology

**DOI:** 10.1002/fsn3.2807

**Published:** 2022-04-19

**Authors:** Sedigheh Mohammadnezhad, Jamshid Farmani

**Affiliations:** ^1^ 185206 Department of Food Science and Technology Faculty of Agricultural Engineering Sari Agricultural Sciences and Natural Resources University Sari Iran

**Keywords:** characterization, chicken skin fat, chicken skin gelatin, extraction condition, solid fat content

## Abstract

Chicken skin is a major byproduct of the poultry industry. This study was undertaken to extract and characterize fat and gelatin from chicken skin. To do this, the chicken skin was wet‐rendered at different temperature–time combinations and the yield and properties of the extracted gelatin and fat were determined. Gelatin and fat were recovered at yield ranges of 0.74%–2.03% and 24.01%–27.91%, respectively. The time and the interaction of time–temperature had a positive effect on gelatin yield (*p* < .05); however, the fat yield was not affected by the extraction condition. Protein, ash, and hydroxyproline content of gelatin and unsaponifiables and free fatty acids contents, peroxide value, and induction period of oxidation of the fat were affected by the extraction condition. Functional and rheological analyses showed chicken skin gelatin gel/solution had a higher bloom value, viscosity, foaming capacity, storage and loss moduli, and melting and gelling points than the commercial bovine gelatin. Oleic (42.13%), palmitic (24.6%), and linoleic (17.53%) acids were the main fatty acids of chicken skin fat. The storage modulus of chicken skin fat was higher than the loss modulus up to 31°C; however, because of a low slip melting point (22.74°C) and solid fat content, it was fluid at room temperature. The findings of this research can be useful in the development of processes for the extraction and application of chicken skin gelatin and fat.

## Graphical abstract

Gelatin (CSG) and fat (CSF) were coextracted from chicken skin at yields of 0.74%–2.03% and 24.01%–27.91%, respectively. Yield, protein, ash, and hydroxyproline content of CSG, and FFA, PV, unsaponifiables, and induction period of oxidation of CSF were affected by extraction conditions. CSG had higher bloom value, viscosity, foaming capacity, G', G'', and melting and gelling points than bovine gelatin, and CSF contained oleic acid as the major fatty acid and showed a crossover point (G''/G' = 1) of 31°C.

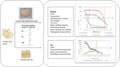

## 1 INTRODUCTION

Chicken skin is one of the main byproducts of the chicken slaughterhouses and portioning industry (Ockerman & Hansen, 1999). It contains about 45% fat (wet basis, Cliche et al., 2003) and about 9% proteins (wet basis, Farmani & Rostammiry, 2015) that make it a potential raw material for the recovery of fat and protein (or their derivatives). Chicken skin fat is composed of about 30% saturated fatty acids, 50% monounsaturated fatty acids, and about 20% polyunsaturated fatty acids (Fallah‐Delavar & Farmani, 2018; Naderi et al., 2016) and several applications, including soap production, biodiesel production (Shi et al., 2013), and use in animal feed formulation (Okur, 2020) and margarine and shortening production (Naderi et al., 2016), have been described for that. According to Bonifer and Froning (1996), proteins of chicken skin include 29% collagen, 10% salt‐soluble proteins, 12% water‐soluble proteins, and 49% other proteins. Chicken skin proteins have been recovered in different forms, including collagen (Cliche et al., 2003), gelatin (Sarbon et al., 2013), protein hydrolyzates (Fallah‐Delavar & Farmani, 2018), and bioactive peptides (Sarbon et al., 2018), of which gelatin is the most popular in the food industry.

Gelatin, which is commonly obtained by partial hydrolysis of collagen, is frequently used as a gelling agent in foods, beverages, medications, drug and vitamin capsules, and cosmetics. Most gelatin is derived from pork skin, pork and cattle bones, or split cattle hides. In this context, poultry gelatin has gained much attention because of some restrictions to the mammalian gelatins like bovine spongiform encephalopathy and foot‐and‐mouth disease concerns related to the bovine gelatin and religious objections to porcine gelatin (Abedinia et al., 2020). Poultry gelatin is obtained from the skin, feet, head, and bones of the bird (Almeida & Caetano, 2013; Rafieian et al., 2015). Taking into account that the chicken meat production was recorded as 88% of overall global poultry meat production in 2020 (FAO, 2021) and skin makes up 15% of the chicken carcass's weight, chicken skin could be regarded as an alternative source of gelatin. As compared to the bovine gelatin, chicken skin gelatin forms stronger hydrogen bondings and contains higher amounts of alpha‐helix and β‐sheet‐type structures. Consequently, chicken skin gelatin has a higher bloom value (355 g vs. 259 g), viscous and elastic modulus values, and melting temperature than bovine gelatin (Mrázek et al., 2019; Sarbon et al., 2013). As a result, valorization of chicken skin for the recovery of value‐added products like gelatin and fat is of great importance.

Various extraction methods, including wet rendering (Lin & Tan, 2017; Sheu & Chen, 2002), dry rendering (Farmani et al., 2016; Lin & Tan, 2017; Sheu & Chen, 2002), microwave rendering (Lin & Tan, 2017; Sheu & Chen, 2002), frying (Sheu & Chen, 2002), and enzyme‐assisted extraction (Fallah‐Delavar & Farmani, 2018), have been used for the recovery of chicken fat from chicken skin. For the recovery of gelatin, enzyme‐assisted extraction is not a choice as the protein hydrolyzates do not normally show gelling properties (Fallah‐Delavar & Farmani, 2018). Dry rendering methods (including microwave and frying techniques) involve the use of higher temperatures (up to 180°C) which can badly affect the quality of fat and proteins (Farmani et al., 2016; Lin & Tan, 2017; Sheu & Chen, 2002). Moreover, the proteinaceous leftover may not be suitable for subsequent gelatin extraction, especially if very high temperatures are used for fat extraction. Wet rendering, on the other hand, involves the use of water and temperatures lower than 100°C (Lin & Tan, 2017; Sheu & Chen, 2002). Accordingly, both fat and gelatin (as an aqueous solution) can be recovered from the chicken skin in a single extraction process. This study discusses the effect of factors involved in the simultanious extraction of gelatin and fat from chicken skin in a wet rendering process. Rheological, physicochemical, and functional properties of the recovered chicken skin gelatin and fat are described as well. Results of this study help the development of a simple process for gelatin and fat extraction from chicken skin.

## 2 MATERIALS AND METHODS

### 2.1 Materials

Chicken skin was bought from a chicken store in Babol, Iran, and washed three times with water. Then, it was ground with a domestic meat grinder and stored at −18°C until use. Commercial bovine gelatin was purchased from Faravari Darooi Gelatin Halal (Qazvin, Iran). All chemicals were purchased from Merck Group (Darmstadt, Germany).

### 2.2 Extraction of gelatin and fat from chicken skin

A frozen ground chicken skin sample was put out from the freezer a few hours before the extraction. Then, 240 ml water was added to 240 g of the chicken skin sample. Fat and gelatin were extracted at different temperatures (80, 90, and 100°C) and times (0.5, 1, and 1.5 h) using a rotary evaporator. The extracted mixture was vacuum filtered and the filtered fraction was poured in a decanter to withdraw the fat phase from the aqueous one. Finally, the fat phases were washed two times with water, decanted, and centrifuged at 8000 × *g* to obtain the chicken skin fat. The aqueous phase containing gelatin was washed five times with equal volumes of n‐hexane, then the remaining n‐hexane was removed using a rotary evaporator (80°C, 0.9 abs bar, 20 min, RV10, IKA, Staufen, Germany). The solution containing gelatin was centrifuged at 10,000 × *g* for 5 min and dried using a freeze dryer (Vaco 2, Zirbus, Bad Grund, Germany).

### 2.3 Yield and physicochemical properties of gelatin and fat

The gelatin or fat yield was calculated as the percentage of gelatin powder or fat obtained from fresh skin. Moisture, ash, fat, protein, and hydroxyproline content of gelatin powder were measured according to the Association of Official Analytical Chemists methods 925.09, 900.02, 922.06, 22.012–22.013, and 990.26, respectively (AOAC, 1995). Moisture, unsaponifiables content, free fatty acids (FFA) content, peroxide value (PV), Lovibond color, induction period of oxidation at 110°C (IP_110_), slip melting point (SMP), and solid fat content (SFC, measured by pulsed nuclear magnetic resonance spectroscopy) of chicken skin fat were determined following the official methods of the American Oil Chemists’ Society (methods Ca 2c–84, Ca 6a‐40, Ca 5a‐40, Cd 8 ‐53, Cc 92‐ e13, Cd 12b‐92, Cc3‐25, and Cd 16–81, respectively, AOCS, 1996).

### 2.4 Gel strength

Solutions of gelatin (6.67 g in 100 g distilled water) were prepared and held at 25°C for 30 min, as described by Almeida and Caetano, (2013). Afterward, the temperature was increased to 65°C in a water bath for 25 min with constant shaking until gelatin was completely dissolved. Then, it was left at 10°C for 16–18 h and then analyzed with a texture profile analyzer (Texture Pro. CT, Brookfield Engineering, USA). The gel strength was determined (at 4 mm depth and speed of 0.5 mm/s) with a cylindrical probe. Texture Expert program was used to analyze the data (Texture Technologies Corp., England, U.K.).

### 2.5 Viscosity

Gelatin solutions were prepared by dissolving 6.67 g of the powder in 100 g of distilled water and heating at 30°C for 30 s. The viscosity of the solutions was measured at 30°C using the spindle No. 1 at speed of 100 rpm using a Brookfield digital viscometer (Model DVII, Brookfield Engineering, USA) (Kim et al., 1994).

### 2.6 Water holding capacity

Briefly, 1 g gelatin powder was dispersed in 50 g distilled water and held at 30°C for 1 h. Then, the mixture was centrifuged at 450 × *g* for 20 min and after removing the supernatant, the centrifuge tube was drained on a filter paper (tilting to an angle of 45°) for 30 min and weighed. Water holding capacity (WHC) was expressed as gram water held by 100 g gelatin (Rafieian et al., 2015).

### 2.7 Oil‐binding capacity

Oil‐binding capacity (OBC) was measured as described above (WHC determination), but sunflower oil (10 g) was used instead of water. OBC was expressed as gram sunflower oil retained by 100 g gelatin powder (Rafieian et al., 2015).

### 2.8 Foaming capacity and foam stability

As described by Cho et al. (2004), 50 ml of gelatin solution (1 g/100 ml) was whipped at 10,000 rpm (Euro Turr ax T20b homogenizer, IKA Labortechnik, Stufen, Germany) for 5 min and the volume of foam was immediately measured using a graduated cylinder. Foaming capacity was defined as the percentage volume increase in the whipped sample. Foam stability was measured as the percentage foam volume remained after standing for 30 min.

### 2.9 Fatty acid composition

Methylation of fat samples was done following the AOCS method Ce 2‐66 (AOCS, 1996). Fatty acid methyl esters (FAME) were identified using a gas chromatograph (model 2550 TG, Teif Gostar Faraz, Tehran, Iran) according to the AOCS method Ce 1e‐91 (AOCS, 1996). A CP Sil 88 column (100 m, 0.25 mm id, and 0.2 µm film thickness; Chrompack, Netherlands) coupled with a flame ionization detector were used to resolve and detect FAMEs. Injector (split ratio of 1:100) and detector temperature was set at 250°C and the column temperature was 175°C. The head pressure of the column was 230 kPa and nitrogen was used as a carrier gas.

### 2.10 Rheological characteristics of chicken skin gelatin and fat

Rheological properties were measured using a controlled‐stress rheometer (MCR 301, Anton‐Paar, GmbH, Austria). Measurements were performed using the parallel‐plate geometry for chicken skin gelatin gels and concentric cylinder geometry for chicken skin fat. Frequency sweep tests (0.1–100 Rad/s) were done in the linear viscoelastic area at 10°C for samples containing 3, 6.67, and 10% gelatin, and at 5°C for fat sample. The temperature sweep tests of gels were done from 50 to 10°C (gelation) and also from 10 to 50°C (melting) at 2°C/min cooling/heating rate and the oscillation frequency of 1 Hz (Sarbon et al., 2013). For analysis of fat samples, they were first melted at 90°C for 5 min, then cooled down to 0°C at a rate of 20°C/min, and finally stored at 5°C for 24 h. The temperature sweep tests of chicken skin fat were performed between 0 and 45°C at a rate of 1°C/min (Naeli et al., 2017). The two main parameters determined in the rheological investigations included storage (G', describing the stored energy and representing the elastic portion) and loss (G'', describing the energy dissipated as heat and representing the viscous portion) modulus. The loss factor (tanδ) which describes the ratio of the two portions (G''/G') of the viscoelastic behavior was calculated as well. The temperature at which G''/G' = 1 (the crossover point) in the cooling or heating cycle of the temperature sweep test was regarded as the gelling or melting point of gelatin gels, respectively.

### 2.11 Statistical analysis

All the measurements were done using a completely randomized factorial design. Results were analyzed using the one‐way or two‐way analysis of variances at the significance level of *p* = .05 by SPSS (IBM SPSS Statistics Ver. 21, New York). The experiments were repeated three times.

## 3 RESULTS AND DISCUSSION

### 3.1 Gelatin and fat yield

In this study, the wet rendering method was used for the simultanious extraction of gelatin and fat from chicken skin waste. The mean gelatin yield of chicken skin was 1.59% on wet weight basis (3.53%, on dry basis, Table 1). Statistical analysis showed that time, as well as the interaction of time and temperature, had positive impacts on the gelatin yield (*p* < .05), but the effect of temperature was insignificant (*p* > .05). Our result was close to the yields (1.74%–4.43%) found by Kim et al. (2012). They illustrated the impact of acid and alkali pretreatments and extraction temperature on the physicochemical characteristics of chicken skin gelatin. The yields obtained here were lower than the values found by Rasli and Sarbon (2015) (9.25%–12.86%, dry basis). They used an alkaline–acid pretreatment for extraction of gelatin from defatted chicken skin which led to a higher gelatin yield. Elsanat et al. (2014) reported chicken skin gelatin yield as 9.93%–24.03% (dry basis) and found that the yield was higher at 2.5% alkaline concentration, skin soaking time of 60 h, extraction temperature of 60°C, and extraction time of 6. Herein, we used a simple extraction method, involving only water, for the extraction of gelatin and fat without any pretreatment of the raw material. The variation in results may be due to the difference in the efficacy of methods, as temperature and extraction time, pH, and pretreatment conditions affect the yield (Rasli & Sarbon, 2015).

**TABLE 1:** Effect of wet rendering condition on yield and properties of gelatin and fat extracted from chicken skin

Temperature (°C)	Wet rendering conditions								
	80			90			100		
Time (h)	0.5	1	1.5	0.5	1	1.5	0.5	1	1.5
Chicken skin gelatin yield and properties^a^									
Gelatin yield (%)	1.15 ± 0.21	1.53 ± 0.18	2.03 ± 0.04	0.74 ± 0.07	1.73 ± 0.04	1.87 ± 0.17	1.30 ± 0.14	1.53 ± 0.04	1.87 ± 0.27
Moisture (%)	3.87 ± 0.45	4.96 ± 0.03	4.25 ± 0.91	4.59 ± 0.43	5.26 ± 0.14	8.59 ± 1.96	3.23 ± 0.19	7.65 ± 2.02	5.67 ± 0.66
Protein (%dry)	96.53 ± 1.41	96.58 ± 5.65	93.93 ± 2.12	93.18 ± 1.00	93.08 ± 2.82	90.22 ± 1.41	93.97 ± 4.24	94.97 ± 4.20	89.30 ± 2.82
Fat (%dry)	1.20 ± 0.00	1.99 ± 0.83	2.85 ± 0.77	4.72 ± 0.20	3.58 ± 0.63	3.97 ± 0.62	2.54 ± 0.70	1.73 ± 0.21	4.24 ± 0.56
Ash (%dry)	2.27 ± 0.07	1.42 ± 0.21	3.22 ± 0.14	2.10 ± 0.35	3.35 ± 0.21	5.82 ± 0.21	3.48 ± 0.17	3.30 ± 1.34	6.47 ± 0.40
Hydroxyproline (%)	4.52 ± 0.07	4.00 ± 0.04	5.17 ± 0.90	4.12 ± 0.88	3.65 ± 0.20	4.45 ± 0.40	3.32 ± 0.55	4.80 ± 0.23	4.17 ± 0.10
Chicken skin fat yield and properties^b^									
Fat yield (%)	26.99 ± 0.47	26.99 ± 0.47	24.05 ± 0.70	26.96 ± 0.51	27.91 ± 0.57	27.08 ± 0.59	25.33 ± 1.88	27.41 ± 0.12	26.66 ± 2.53
Moisture (%)	0.15 ± 0.07	0.11 ± 0.02	0.11 ± 0.01	0.10 ± 0.01	0.12 ± 0.01	0.11 ± 0.02	0.10 ± 0.01	0.12 ± 0.01	0.10 ± 0.01
Unsaponifiables (%)	0.24 ± 0.03	0.19 ± 0.07	0.28 ± 0.05	0.32 ± 0.08	0.43 ± 0.09	0.34 ± 0.07	0.24 ± 0.07	0.30 ± 0.03	0.36 ± 0.05
Free fatty acids (%)	0.48 ± 0.00	0.52 ± 0.03	0.55 ± 0.01	0.50 ± 0.01	0.52 ± 0.02	0.54 ± 0.02	0.49 ± 0.02	0.51 ± 0.01	0.54 ± 0.02
PV (meq/kg)	4.92 ± 0.84	8.06 ± 1.02	9.38 ± 2.08	4.66 ± 0.15	8.44 ± 0.26	9.84 ± 1.88	4.91 ± 90	7.55 ± 0.98	9.52 ± 1.66
IP_110_ (h)	1.11 ± 0.39	0.81 ± 0.73	0.66 ± 0.49	0.42 ± 0.00	0.35 ± 0.04	0.52 ± 0.01	0.55 ± 0.01	0.49 ± 0.12	0.37 ± 0.02
Lovibond red color	1.01 ± 0.03	1.10 ± 0.03	0.90 ± 0.01	0.80 ± 0.01	0.80 ± 0.03	0.90 ± 0.02	0.85 ± 0.02	0.90 ± 0.01	1.00 ± 0.02
Lovibond yellow color	11.00 ± 1.00	11.00 ± 1.41	12.00 ± 1.41	12.50 ± 3.53	12.50 ± 0.07	12.50 ± 2.05	11.50 ± 0.70	12.00 ± 2.82	9.50 ± 0.70

Abbreviations: IP_110_, induction period of oxidation at 110°C; PV, peroxide value.

^a^ Extraction time, and the interaction of extraction time and temperature, had positive effects on gelatin yield. The interaction of extraction time and temperature showed a significant effect on the protein content. Extraction time and temperature, and their interaction, had positive effects on the ash content. The effect of the interaction of extraction time and temperature on the hydroxyproline content of the gelation was significant (all at *p* < .05).

^b^ Extraction temperature had a significant positive effect on the content of unsaponifiables, free fatty acids, and PV. The IP_110_ of chicken skin fat was negatively affected by the extraction temperature (all at *p* < .05).

The yield of chicken skin fat ranged from 24.01% to 27.91% (26.60% on average). The results showed that none of the time, temperature, or interaction between them had a significant effect on the yield (*p* > .05). The yield of chicken skin fat reported in this study was similar to those obtained by the wet rendering of chicken skin (23.3%, Lin & Tan, 2017) but lower as compared to the yields found for enzyme‐assisted extraction of chicken skin fat (30.4%–35.85%, Fallah‐Delavar & Farmani, 2018). The differences between the results may be explained by the diverse efficacy of different extraction methods.

### 3.2 Composition of the chicken skin gelatin

The composition of the obtained chicken skin gelatin is shown in Table 1. The contents of protein and ash in the gelatin are mainly dependent on the extraction procedure. The protein and ash contents of the chicken skin gelatin were 89.30%–96.58% (82.94%, wet basis on average) and 1.42%–6.47% (3.12%, wet basis on average) on dry basis, respectively. Neither the extraction time nor the temperature showed a significant effect on the protein content of the gelatin; however, the interaction of time and temperature showed a significant effect (*p* < .05). In terms of the content of ash, both the extraction time and temperature, as well as their interaction, had positive effects on the ash content of gelatin (*p* < .05). Sarbon et al. (2013) reported the protein content of the gelatin extracted from chicken skin as 80.73% which was lower than what is found here. The ash content of the gelatin extracted from chicken skin in this study was lower than that reported for gelatin extracted using acid pretreatment from chicken deboner residue (4.41%, Rafieian et al., 2015), and higher than that obtained by Sarbon et al. (2013) for chicken skin (0.32%). In addition to the pretreatments and extraction method, the ash content of gelatin varies based on the type of gelatin source. Almeida and Caetano (2013) found that the ash content of gelatin obtained from metatarsus was more than six times higher than that extracted from tendons and skins of chicken feet. According to the specification described by the Joint FAO/WHO Expert Committee on Food Additives (JECFA, 2004), the content of ash in dried gelatin should not be more than 2%. As the ash content obtained in this study was higher, deionization of the gelatin solution should be adopted as a pretreatment (Schrieber & Gareis, 2007).

The moisture and fat content of the gelatin were 3.87%–8.59% and 1.20%–4.72% (dry basis), respectively. These two characteristics are mainly affected by the separation efficacy of the purification and drying methods rather than the extraction technique. However, these values were lower compared to those found by other researchers (Bueno et al., 2011; Sarbon et al., 2013). The values found in this study were per the JECFA standard (JECFA, 2004), which determined the maximum loss on drying (which represents moisture and volatile matters) of edible gelatin as 18%.

### 3.3 Hydroxyproline content of gelatin

Hydroxyproline is a major part of the collagenous protein and is specific for gelatin. It comprises roughly 13.5% of mammalian collagen; accordingly, it can be used for the quantitative and qualitative evaluation of gelatin (Schrieber & Gareis, 2007). The extraction temperature and time had no significant effects on the hydroxyproline content of the gelatin; however, the effect of the interaction of time and temperature was significant (*p* < .05). The mean hydroxyproline content of chicken skin gelatin was 4.24% (Table 1). According to Tümerkan et al. (2019), chicken skin contained 0.63% hydroxyproline, which rose to 6.4% in the extracted gelatin obtained using an alkaline–acid pretreatment. The hydroxyproline content of commercial bovine and porcine gelatin was found to be 10.5 and 6.5%, respectively, as reported by Jamilah and Harvinder (2002).

### 3.4 Gel strength

Gel strength or bloom value is defined as the needed mass or force in reaching the necessary depression (a depth of 4 mm) of the surface of a 6.67% gelatin gel. Gelatins are categorized as low bloom (50–100 g), medium bloom (100–200 g), and high bloom (200–300 g) depending on their bloom value (Schrieber & Gareis, 2007). In this research, the bloom value of gelatin obtained from the chicken skin gelatin was 291 ± 16.90 g. Accordingly, chicken skin gelatin can be regarded as a high bloom gelatin. The bloom value of the extracted gelatin was higher than that of the commercial bovine gelatin (250 ± 7.07 g, *p* <.05). Sarbon et al. (2013) reported a higher bloom value (355 g) for chicken skin gelatin. This may be because they had used different extraction treatments in their study, and their gelatin had a higher hydroxyproline content (12.13%). In another study, Rahman & Jamalulail (2012) found the gel strength of chicken feet gelatin as 264.33 g. Chicken skin gelatin obtained in this study was found to have better gel strength in comparison to gelatins extracted from shortfin scad (177 g), tilapia (181 g, Grossman & Bergman, 1992), and sin croaker (125 g, Cheow et al., 2007). The differences in strength of various gelatins could be illustrated by differences in their amino acid composition, collagen ratio, method of extraction, and the inherent characteristics of collagens from different species (Badii & Howell, 2006).

### 3.5 Viscosity

Gels formed from gelatin solutions with high viscosity are extensible and hard, and gels formed from low‐viscosity gelatin are weak and have a brittle texture. Standard viscosity is measured primarily for a 6.67% gelatin solution (Schrieber & Gareis, 2007). In this research, the viscosity of chicken skin gelatin and commercial bovine gelatin was found to be 22.71 ± 0.04 and 21.75 ± 0.04 cP, respectively. In a similar study, Bichukale et al. (2018) reported the viscosity of chicken skin gelatin in the range of 3.83–5.53 cP at 60°C. Rafieian et al. (2015) reported the viscosity of gelatin obtained from chicken deboner residue as 5.85 cP at 60°C.

### 3.6 Rheological properties of gelatin gels

**FIGURE 1:**
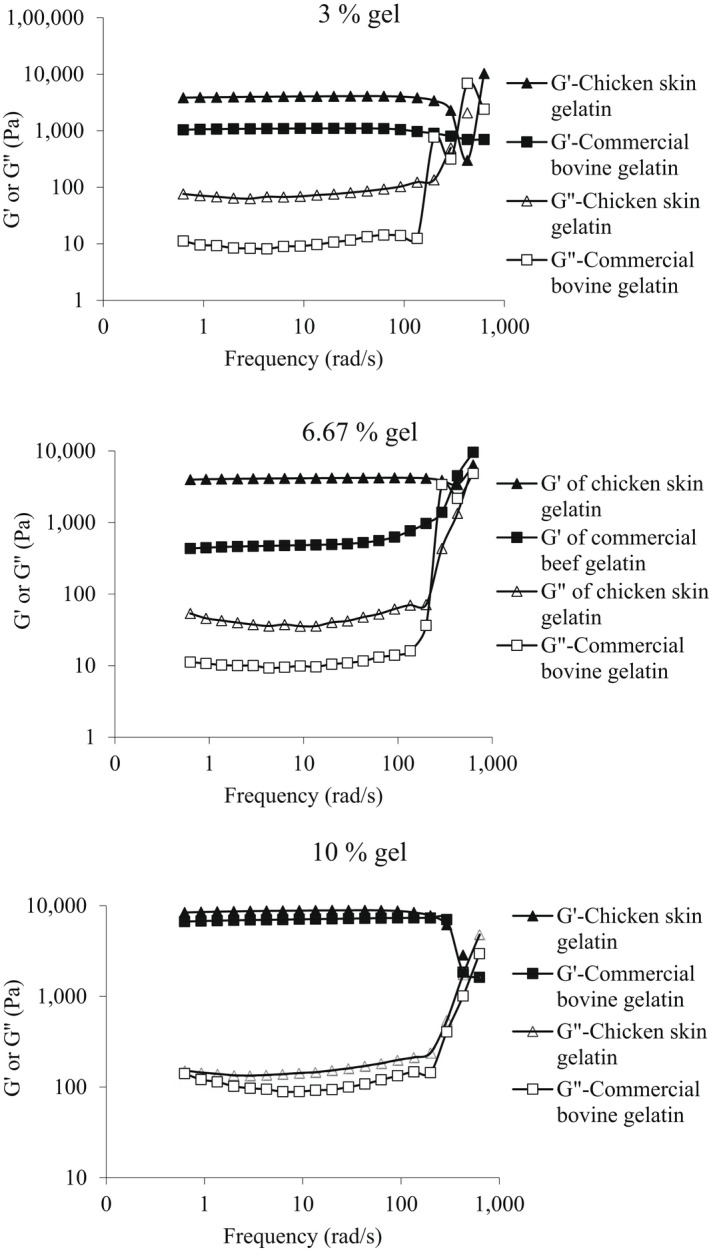
Effect of concentration (3, 6.67, and 10% (w/v)) on viscoelastic properties of chicken skin and commercial bovine gelatins in a frequency sweep test at constant temperature (10°C)

Rheograms of the frequency sweep tests are illustrated in Figure 1. The values of storage modulus (G') were higher than the values of the loss modulus (G") at all the concentrations (*p* < .05), showing the solid‐like behavior of the gels. Furthermore, values of both G' and G" grew with the increase in gelatin concentration (*p* < .05). The G' value of the extracted gelatin was higher than that of the commercial bovine gelatin, probably due to the weaker intermolecular interaction of commercial bovine gelatin in comparison to the chicken skin gelatin. Similar G' and G" values had also been reported by Sarbon et al. (2013). The storage modulus of both gels (made from commercial bovine gelatin or chicken skin gelatin) showed a slight frequency dependency at 3% (w/v) concentration, while at higher concentrations (6.67 and 10% (w/v)), no frequency dependency was observed. A similar result was also found for the storage modulus of gels made from cod skin gelatin which showed much less frequency dependency at higher gelatin concentrations (Gilsenan & Ross‐Murphy, 2000).

**FIGURE 2:**
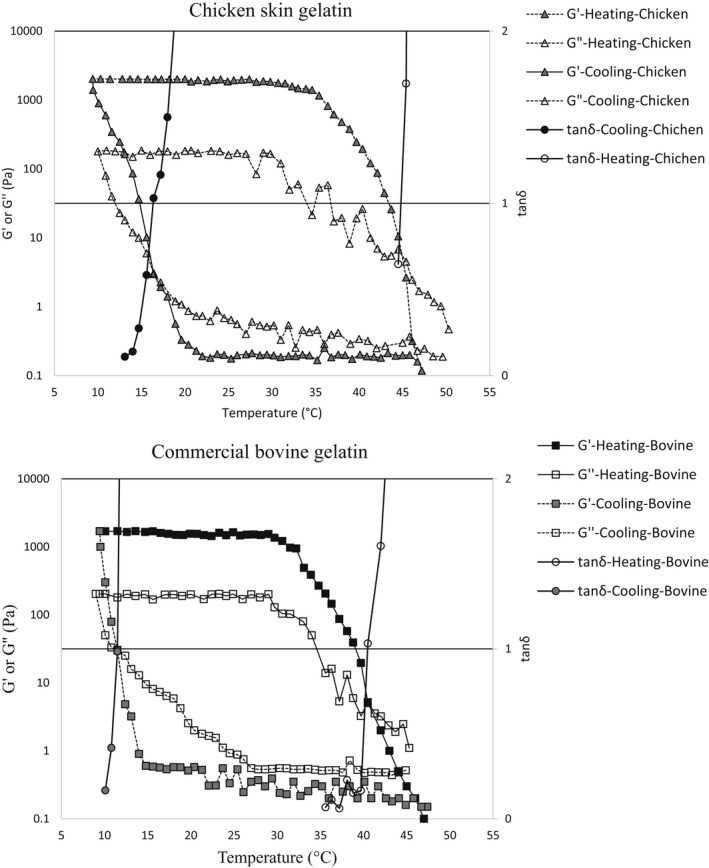
Viscoelastic properties (G' and G" values) of the chicken skin gelatin and the commercial bovine gelatin in temperature sweep tests (including cooling from 50 to 10°C and subsequent heating from 10 to 50°C)

Figure 2 compares the dynamic viscoelastic profile of chicken skin gelatin and the commercial bovine gelatin during both cooling from 50 to 10°C and heating from 10 to 50°C in temperature sweep tests. In the cooling tests, G' curves rose sharply when the temperature decreased from 50°C to 30–35°C (30°C for the bovine and 35°C for the chicken skin gelatin). This indicates a rapid formation of junction zones and strong reinforcement of the gel network and the corresponding increase in the amount of stored energy. Chicken skin gelatin showed higher G' values at all temperatures compared to the commercial bovine gelatin which is indicative of its enhanced ability to refold into a triple helix (Gómez‐Guillén et al., 2002). The higher G' value of chicken skin gelatin, as compared to the commercial bovine gelatin, also indicates its higher heat stability during both the cooling and heating cycles. Generally, high G' values and thermostability are mainly related to amino acid composition, with hydroxyproline playing a unique role in stabilizing the triple helix. Gómez‐Guillén et al. (2002) also correlated the thermal stability of gelatin to the amount and stability of proline‐rich regions in collagen and gelatin molecules, which vary among gelatins from different sources. The maximum values of G' and G" for chicken skin gelatin (2030 and 180 Pa, respectively) and commercial bovine gelatin (1700 and 203 Pa, respectively) were found to be much lower than the values found by Sarbon et al. (2013).

**FIGURE 3:**
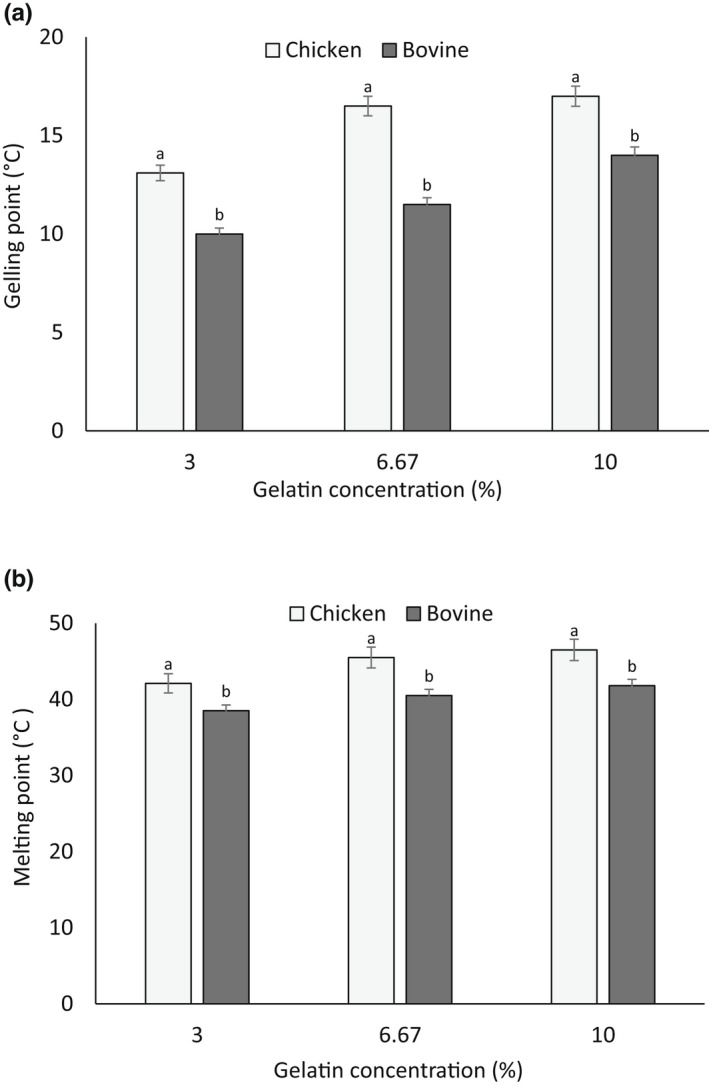
The effect of gelatin concentration on (a) gelling and (b) melting point of chicken skin gelatin and commercial bovine gelatin

The gelling and melting temperatures of the 6.67% (w/v) chicken skin gelatin were 16.5 and 45.5°C, respectively. Meanwhile, the commercial bovine gelatin showed a gelling and melting point of 11.5 and 40.5°C, respectively (Figure 3a and b). In a similar study done by Sarbon et al. (2013), the gelling and melting point of chicken skin gelatin were found to be 24.88 and 33.57, respectively. Figure 3a and b compares the effects of gelatin concentrations on the gelling and melting temperature of chicken skin and the commercial bovine gelatin. The gelling temperature of both chicken skin and commercial bovine gelatins was concentration dependent and increased when the concentration increased, probably due to the formation of higher amounts of hydrogen bondings at higher gelatin concentrations (Sarbon et al., 2013). The gelling point of chicken skin gelatin was higher than that of the bovine gelatin at all concentrations (*p* < .05). The difference in the gelling point of gelatins may be due to the intrinsic differences in the protein structure. Similarly, chicken skin gelatin melted at a higher temperature than the commercial bovine gelatin and the melting point of gels increased with the increase in gelatin concentration, as shown in Figure 3b (*p* < .05). The effect of gelatin concentration on the gelling and melting point of gels has been previously documented (Sarbon et al., 2013). The difference in thermostability between chicken skin and commercial bovine gelatin may be attributed to the higher proline and hydroxyproline content of chicken skin gelatin (Gómez‐Guillén et al., 2002).

### 3.7 Water holding capacity of gelatin

WHC refers to the ability of the protein to imbibe water and retain it within a protein matrix against the gravitational force. WHC of chicken skin gelatin (1553.00 ± 110.30 g/100 g) was lower than the commercial gelatin (2380.00 ± 84.80 g/100 g, *p* < .05). These values were much higher than that reported by Rafieian et al. (2015) for gelatin extracted from chicken deboner residue (859.00 ± 60.02 g/100 g). These values were also much higher as compared to the previously reported results by Shyni et al. (2014) for gelatin from the skins of skipjack tuna, dog shark, and rohu. Intrinsic factors affecting WHC of food proteins include size, shape, amino acid composition, protein conformation, surface hydrophobicity/polarity, and the presence of lipids and carbohydrates on the surface of protein particles (Schrieber & Gareis, 2007).

### 3.8 Oil‐binding capacity of gelatin

OBC is a functional property that is closely related to texture and other food properties. This property is influenced by protein source, processing conditions, additive composition, particle size, and temperature (Schrieber & Gareis, 2007). This study revealed that chicken skin gelatin had a higher OBC (164.5 ± 6.36 g of oil/100 g) than the commercial bovine gelatin (144.5 ± 4.94 g of oil/100 g). These values were higher, as compared to the mean values reported by Rafieian et al. (2015) for gelatin extracted from chicken deboner residue (67.26 ± 7.97 g of oil/100 g). Ninan et al. (2014) reported that the difference in OBC may be due to the variation in the amount and type of nonpolar residues of proteins and also the degree of exposure of these hydrophobic residues, which bind to the hydrocarbon side chain of oil.

### 3.9 Foaming properties of gelatin

Proteins are the main surface‐active agents in food products and play a crucial role in air entrapment by decreasing surface tension at the air–liquid interface (Schrieber & Gareis, 2007). The foaming capacity (% volume increase) of chicken skin gelatin (176.5 ± 2.12) found in this study was higher than that of the commercial bovine gelatin (152.0 ± 2.82, *p* < .05). The values obtained here were lower than those reported for porcine skin gelatin (290 ml/100 ml) and shark cartilage gelatin (260 ml/100 ml) (Cho et al., 2004), but were higher than that found for bovine gelatin (93 ml/100 ml) by Nhari et al. (2011).

The stability of foam made using the chicken skin gelatin was 80.28 ± 6.80%, which was not significantly different from that of the foam made using the commercial bovine gelatin (82.28 ± 4.60%, *p* > .05). It has been suggested that reduced foam stability may be due to the aggregation of proteins which interfere with interactions between them and water needed for foam formation (Cho et al., 2004). Foaming properties of proteins are influenced by protein source and composition, intrinsic properties of proteins, and protein's conformation in solution and at the air/water interface (Schrieber & Gareis, 2007).

### 3.10 Physicochemical properties of fat

Chicken skin fat contained 0.11% moisture on average (Table 1), which was much lower than that reported by Sheu and Chen (2002) and Farmani et al. (2016) (1.43% and 0.9%–1.07%, respectively). Moisture can be considered as an impurity and higher moisture content can lead to increased fat hydrolysis and refining loss (O'Brien, 2008). The moisture content of fat samples represents the efficacy of separation techniques used and was not affected by the extraction conditions (*p* < .05).

Pigments, sterols, hydrocarbons, and other compounds that do not saponify with alkali are called unsaponifiables. In this study, the amount of unsaponifiables of chicken skin fat was between 0.19% and 0.43% (Table 1). Feddern et al. (2010) reported a higher amount of unsaponifiables (1.9%) for chicken skin fat. The unsaponifiables content of chicken skin fat extracted by the enzyme‐assisted extraction method was reported as 1.15% (Fallah‐Delavar & Farmani, 2018), which was higher than those reported in this work. The variation in the unsaponifiables content reported in different works is due to the fact that extraction method and condition have a great influence on the amounts of materials extracted. According to the results obtained here, extraction temperature had a significant effect on the content of unsaponifiables (*p* < .05).

The FFA content and PV of fats depend on the lipolytic activity of the starting materials as well as the extraction method (Farmani et al., 2016; Lin & Tan, 2017). In this study, the FFA content and PV of chicken skin fat were in the range of 0.48%–0.55% and 4.91–9.84 meq/kg, respectively, being higher at higher extraction times (*p* < .05, Table 1). The FFA results match those reported by Sheu and Chen (2002, <1%), Feddern et al. (2010, 0.65%), Zhang et al. (2013, 0.66%–0.88%), Farmani and Rostammiry (2015, 0.62%), and Farmani et al. (2016, 0.16%–0.70%) for chicken fat. The PV of chicken skin fat was in agreement with other studies reported for chicken skin fat (Sheu & Chen, 2002 (4.3–6.4 meq/kg), Feddern et al., 2010 (2.14 meq/kg), and Zhang et al., 2013 (3.4–7.1 meq/kg)).

Oxidative stability index, measured as IP_110_, is an indicator of the tendency of oil to oxidation. The IP_110_ of chicken skin fat was 0.37–1.11 h, which was negatively affected by the extraction temperature (Table 1, *p* < .05). Naderi et al. (2016) also reported a value of 0.97 h for chicken skin fat. The low IP_110_ of the crude chicken skin fat limits its application in shortening production since most standards of specification of shortenings (like Iran's national standard, INSO, 2015) require an IP_110_ larger than 25 h. Refining, bleaching, and deodorization of the chicken skin fat and adding antioxidants may improve oxidative stability.

The color of fat is mainly related to the presence of carotenoid pigments (yellow–red color) or chlorophyll in the vegetable oil (O'Brien, 2008). The red and yellow indices of chicken skin fat were in the range of 0.8–1.1 and 9.5–12.5, respectively, which were not affected by the extraction condition (Table 1). This was in agreement with the result reported for chicken breast skin fat by Sheu and Chen (2002).

### 3.11 Fatty acid composition of chicken skin fat

The fatty acid composition of chicken skin fat is shown in Table 2. Five important fatty acids of chicken skin fat were found to be oleic, palmitic, linoleic, stearic, and palmitoleic acids. The highest level belonged to oleic acid (42.13%), which makes chicken skin fat a good source of monounsaturated fatty acids. It also contained a significant amount of palmitoleic acid which makes it unique among the edible oils. The content of palmitic acid, as the main saturated fatty acid, was 24.62% and the content of other saturated fatty acids such as stearic acid was lower than 6.5%. Totally, chicken skin fat contained 31.53% saturated fatty acids, which is very low, as compared to other common animal fats like lard and tallow (Farmani & Rostammiry, 2015). We have discussed the fatty acid composition of chicken fat elsewhere (Farmani & Rostammiry, 2015).

**TABLE 2:** Fatty acid composition of the extracted chicken skin fat

Fatty acid	Content (%)
14:0	0.69 ± 0.10
16:0	24.62 ± 1.54
16:1 *n*−7	5.65 ± 0.44
18:0	6.22 ± 0.29
18:1 *n*−9	42.13 ± 1.52
18:2 *n*−6	17.53 ± 0.36
18:3 *n*−3	0.96 ± 0.03
20:1 *n*−9	0.42 ± 0.04
SFA	31.53

Abbreviation: SFA, total saturated fatty acids.

### 3.12 Slip melting point of fat

The SMP of chicken skin fat was found to be 22.74°C (Table 3). According to Farmani and Rostammiry (2015) and Naderi et al. (2016), the SMP of chicken fat was 25.8°C and 27.5°C. Similar results were also found by Arnaud et al. (2004). SMP of the extracted fat is much lower than that of tallow (45–48°C), lard fat (31–33.5°C), and palm oil (35.5–45°C), which is because of the lower content of saturated fatty acid and high melting point TAGs of chicken fat (O'Brien, 2008).

**TABLE 3:** Slip melting point and solid fat content of chicken skin fat

Slip melting point (°C)	Solid fat content (%)						
	0°C	5°C	10°C	20°C	30°C	35°C	40°C
22.74 ± 0.07	28.74 ± 0.28	26.00 ± 0.48	18.65 ± 0.07	5.95 ± 0.07	1.90 ± 0.14	0.60 ± 0.13	0.15 ± 0.07

### 3.13 Solid fat content

The SFC of chicken skin fat at various temperatures is shown in Table 3. Our results were in agreement with those found by Arnaud et al. (2004) and Farmani and Rostammiry (2015). The low SFC of chicken fat at 20 to 25°C leads to its semi‐liquid state at room temperature. Chicken fat has lower SFC than lard fat tallow, and even palm oil at all temperatures (Farmani & Rostammiry, 2015).

### 3.14 Rheological Properties of fat

**FIGURE 4:**
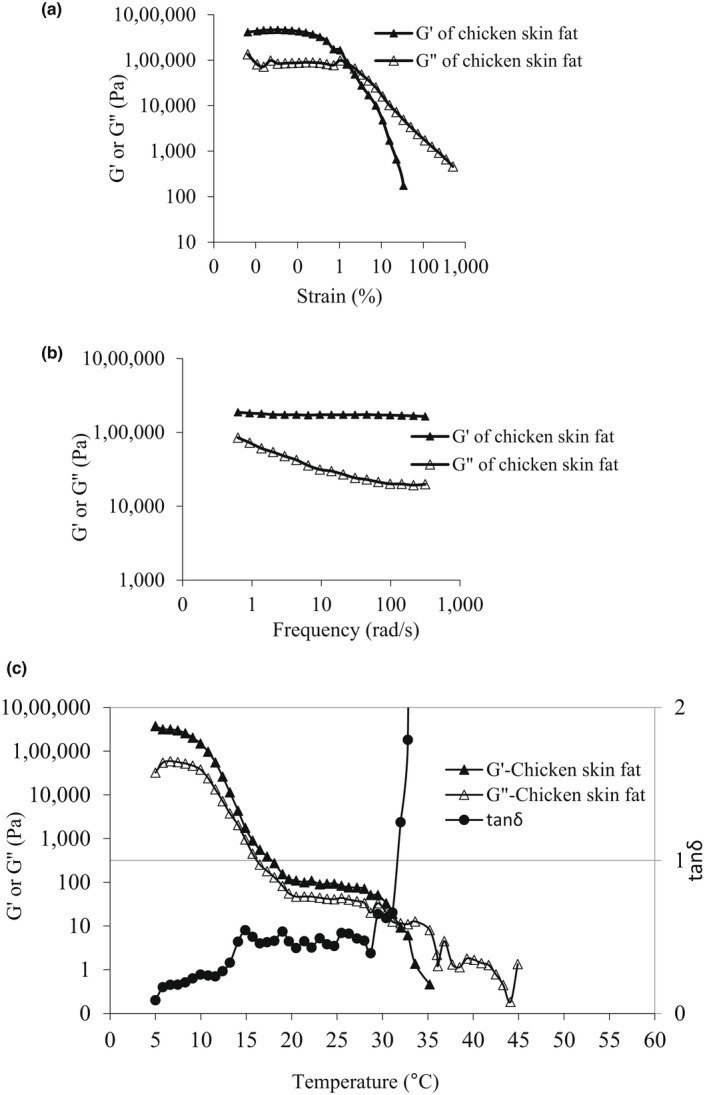
Viscoelastic properties of chicken skin fat. (a) strain sweep test; (b) frequency sweep test; and (c) temperature sweep test

As shown in Figure 4a, the strain sweep rheogram of chicken skin fat could be categorized into two distinct areas: a linear viscoelastic area (LVE), where both G' and G" are constant, and a nonlinear area, where both G' and G" began to drop with the increase in strain (large deformations). G' modulus of chicken skin fat was higher than its G" value in the LVE. Accordingly, the fat sample had a solid‐like behavior within the LVE. Chicken skin fat had a short limiting value of strain (γ_1_) at the linear viscoelastic region, indicating a short LVE limit and lower stability of fat at strains under the γ_1_ amplitude. As shown in Figure 4a, a crossover occurred (at 1.55% strain) and the G" curve dominated the G' curve, showing a liquid‐like behavior of the sample.

In frequency sweep measurements, the G' value of chicken fat was higher than its G" at all frequencies (Figure 4b). Chicken fat showed approximately constant G' value at different frequencies which is indicative of the fat tendency to protect its original shape and form. Frequency sweep could be utilized to determine the spreadability of plastic fats. For this purpose, the frequency where G' and G" crossover reflects the spreadability of fat. The higher the frequency at which crossover occurs, the more spreadable is the fat (O'Brien, 2008). According to this study, the solid‐like behavior of chicken skin fat (higher G') was predominant in the frequency sweep test and had little change at the tested frequency region.

In temperature sweep tests (Figure 4c), both G' and G" dropped with the increase in temperature. There was a sharp fall in G' and G" of chicken fat as the temperature rose from 5 to 20°C (near to the SMP, 22.74°C). After that, both the moduli decreased gradually. The crossover of the G' and G" curves occurred at 31°C (Figure 4c). At the crossover point, G' and G" are equal and tanδ=1. The tanδ is a useful parameter to describe the viscoelastic properties of food samples. The increase in tanδ value to more than 1 means the predominance of viscous nature and vice versa (Lee & Foglia, 2000). In the temperature sweep test, tanδ was lower than 1 at the temperature range of 5–31°C, and after this region, its value was greater than 1, which illustrates the dominance of the viscous nature of the fat.

Natural fats are a mix of different TAGs with different melting points. Accordingly, such materials show a melting range instead of a melting point (Mahjoob et al., 2018). The SMP is a measure of the start of the melting range. At the SMP of chicken skin fat (22.74°C), the SFC of the fat was about 4% (Table 3) and the elastic behavior was still predominant (Figure 4c). At the crossover point (31°C), SFC was lower than 1.9%, and after that chicken fat indicated liquid‐like or viscous behavior, as the G" predominated the G'. Accordingly, it can be concluded that the complete melting of chicken skin fat occurs at temperatures higher than 31°C.

## 4 CONCLUSIONS

To sum up, gelatin and fat could be extracted from the chicken skin using a simple water extraction procedure. Extraction conditions affected the gelatin yield, the content of protein, ash, and hydroxyproline of gelatin, and unsaponifiables and FFA contents, PV, and IP_110_ of the fat. Chicken skin gelatin showed higher viscosity, foaming capacity, bloom value, and storage modulus than the commercial bovine gelatin. Chicken skin fat contained oleic and palmitic acids as the major unsaturated and saturated fatty acids, respectively. The melting point of chicken fat was 22.74°C. The rheological measurements of chicken fat indicated that the value of G' was higher than G" up to 31°C. Based on the results of the present study, gelatin and fat from chicken skin can be considered as a valuable byproduct from the poultry industry.

## CONFLICT OF INTEREST

No conflict of interest is declared by the authors.

## ETHICAL APPROVAL

This study does not involve any human or animal testing.

## Data Availability

The data that support the findings of this study are available on request from the corresponding author. The data are not publicly available due to privacy or ethical restrictions.
